# Global Identification and Characterization of Transcriptionally Active Regions in the Rice Genome

**DOI:** 10.1371/journal.pone.0000294

**Published:** 2007-03-14

**Authors:** Lei Li, Xiangfeng Wang, Rajkumar Sasidharan, Viktor Stolc, Wei Deng, Hang He, Jan Korbel, Xuewei Chen, Waraporn Tongprasit, Pamela Ronald, Runsheng Chen, Mark Gerstein, Xing Wang Deng

**Affiliations:** 1 Department of Molecular, Cellular, and Developmental Biology, Yale University, New Haven, Connecticut, United States of America; 2 National Institute of Biological Sciences, Zhongguancun Life Science Park, Beijing, China; 3 Peking-Yale Joint Research Center of Plant Molecular Genetics and Agrobiotechnology, College of Life Sciences, Peking University, Beijing, China; 4 Department of Molecular Biophysics and Biochemistry, Yale University, New Haven, Connecticut, United States of America; 5 Genome Research Facility, NASA Ames Research Center, Moffett Field, California, United States of America; 6 Bioinformatics Laboratory, Institute of Biophysics, Chinese Academy of Sciences, Beijing, China; 7 Department of Plant Pathology, University of California, Davis, California, United States of America; 8 Eloret Corporation, Sunnyvale, California, United States of America; University of California, Davis, United States of America

## Abstract

Genome tiling microarray studies have consistently documented rich transcriptional activity beyond the annotated genes. However, systematic characterization and transcriptional profiling of the putative novel transcripts on the genome scale are still lacking. We report here the identification of 25,352 and 27,744 transcriptionally active regions (TARs) not encoded by annotated exons in the rice (*Oryza. sativa*) subspecies *japonica* and *indica*, respectively. The non-exonic TARs account for approximately two thirds of the total TARs detected by tiling arrays and represent transcripts likely conserved between *japonica* and *indica*. Transcription of 21,018 (83%) *japonica* non-exonic TARs was verified through expression profiling in 10 tissue types using a re-array in which annotated genes and TARs were each represented by five independent probes. Subsequent analyses indicate that about 80% of the *japonica* TARs that were not assigned to annotated exons can be assigned to various putatively functional or structural elements of the rice genome, including splice variants, uncharacterized portions of incompletely annotated genes, antisense transcripts, duplicated gene fragments, and potential non-coding RNAs. These results provide a systematic characterization of non-exonic transcripts in rice and thus expand the current view of the complexity and dynamics of the rice transcriptome.

## Introduction

Efforts to generate sequence from the rice (*Oryza sativa*) genome as a model for the Gramineae, the group that includes all major cereal crops, has produced draft sequences of the *japonica* and *indica* subspecies [1]–[3]. The current, map-based *japonica* genome sequence assembly covers over 95% of the genome [4]–[7]. These sequences have been subjected to extensive annotation using *ab initio* gene prediction, comparative genomics, and a variety of other computational methods [1]–[8]. As such, our understanding of the rice genome is largely confined to the state-of-the-art in computational gene prediction and annotation. Improvement of rice genome annotation is progressing rapidly along several lines of analysis. Extensive expressed sequence tags (ESTs) and full-length cDNA (FL-cDNA) sequence data have been incorporated to improve the predicted gene structure [7]–[9]. Databases of plant repeat sequences are being expanded and refined to mask open reading frames (ORFs) associated with transposable elements (TEs) [8], [10]. On the other hand, application of various transcriptomic approaches in rice [9], [11]–[13], including genome tiling microarray analysis [14], [15], has documented many novel transcripts that have yet to be incorporated into genome annotation.

Genome tiling arrays are a recent advances in microarray technology that involve the representation of a target genome by a virtual ‘tile path’ consisting of oligonucleotide probes [16], [17]. The probes are synthesized or immobilized on glass slides at high feature densities so that even complex genome sequences can be accommodated within a manageable number of arrays [18]–[20]. Hybridization of the tiling arrays with fluorescence-labeled targets derived from various RNA sources generates signals that can be analyzed to identify transcribed regions of the genome at a resolution roughly equal to the average distance between neighboring probes [15], [20]–[25].

Because genome tiling arrays provide end-to-end coverage of the target genome and measure transcriptional activity from multiple probes, they are capable of detecting the transcriptome in a comprehensive and relatively unbiased way. Thus, in the context of rice genome annotation, tiling array analysis can be utilized to verify predicted gene models and to identify novel transcription units [14], [15]. For example, of the 43,914 annotated non-TE gene models from *indica* [3], transcription of 35,970 (82%) was detected by tiling array analysis [15]. Consistent with results from tiling analysis in other model organisms [20]–[25], significant transcriptional activities were detected outside of the annotated exons of the rice genome [14], [15]. A conservative scoring of *indica* tiling array data identified 5,464 unique transcriptionally active regions (TARs) in the intergenic regions [15].

In the present study, we performed systematic searches and characterization for TARs in the rice genome, taking advantage of the parallel *japonica* and *indica* genome tiling data. We identified 39,018 and 42,470 TARs from *japonica* and *indica*, respectively. About two thirds of the TARs, were detected at corresponding locations in the two rice subspecies and do not intersect with annotated exons (referred to as non-exonic TARs). We verified transcription of the *japonica* non-exonic TARs by using a re-array in which each TAR and gene model was represented by five probes to profile 10 different rice tissue types. The re-array results were consistent with other experimental evidence and validated >83% non-exonic TARs detected in by the tiling array. Through genome-wide bioinformatic characterization, we assigned approximately 80% of the non-exonic TARs to various putative functional or structural elements of the rice genome.

## Results and Discussion

### Identification of TARs from rice full-genome tiling arrays

Our characterization of the *japonica* transcriptome started with custom full-genome tiling microarrays containing 12,254,374 36-mer oligonucleotide probes, with an average space of 10 nucleotides (nt) between adjacent probes. The probes tile both DNA strands of the non-repetitive sequences of the genome and were synthesized in a set of 32 arrays [15], [24], [26]. The tiling arrays were hybridized to a mixture of cDNA targets derived from four rice tissues or organs to optimize transcript representation (see Materials and Methods). Transcription of the annotated gene models was scored using a previously described method [24] and the results are shown in Figure S1. It is note worth that the PASA gene models (see below), which were improved by incorporation of EST and FL-cDNA information, were detected at higher rate than other gene models, attesting to the accuracy of tiling array detection.

**Figure 1:**
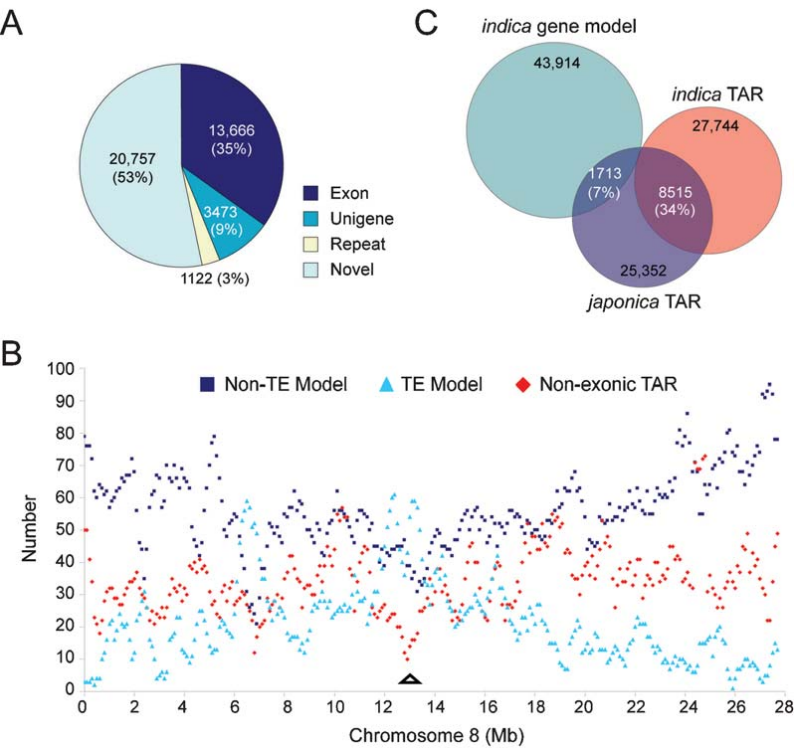
Characterization of *japonica* TARs. (A) Classification of all identified TARs based on known transcriptional evidence and sequences of known origin. Exon, exons of annotated gene models; Unigene, rice Unigenes in GenBank; Repeat, plant repetitive sequences and organelle insertions. (B) Density of different sets of transcription units along Chromosome 8. The number of transcription units was calculated in 500 Kb-sliding-windows with a 100 Kb step. Position of the centromere is indicated by the triangle. (C) Venn diagram showing the comparison of *japonica* non-exonic TARs with *indica* transcription units. Two independent comparisons were made. First, the *japonica* non-exonic TARs were compared with annotated *indica* gene models. Second, the *japonica* non-exonic TARs were compared with *indica* non-exonic TARs, which were identified outside of gene models from tiling array analysis of the *indica* genome. Shaded areas indicate *japonica* TARs that overlap with *indica* gene models or non-exonic TARs.

We next scored genome-wide transcription blind to the annotated gene models and described a transcriptome consisting of 39,018 unique TARs (see Materials and Methods). Of these, 13,666 (35%) intersected with annotated exons (exonic-TAR; Figure 1A). Comparison of the corresponding sequences of non-exonic TARs to plant repeat databases yielded 748 hits (E≤e^−10^). Comparing the non-exonic TARs with rice organelle genome sequences, which have inserted into and contribute to ∼0.4% of the nuclear genome [7], revealed 136 and 238 matches (>95% sequence identity) to the mitochondrial and chloroplast genomes, respectively. Thus, only 1122 (4%) non-exonic TARs are encoded by repeats (Figure 1A). Not surprisingly, distribution of the non-exonic TARs along the chromosomes indicates that the TE-abundant centromere regions are depleted of TARs (Figure 1B and S2).

To facilitate comparison of TARs between *japonica* and *indica* rice, we re-analyzed the previously reported *indica* genome tiling data using the same method reported in the present study. Our re-analysis identified 42,470 *indica* TARs, of which 27,744 (65%) were non-exonic (Figure S3), significantly extending the previous report of 5464 TARs in the intergenic regions [15]. The percentage of TARs intersecting with exons is the same (35%) in *japonica* and *indica*, implying that annotated exons account for approximately one third of the overall transcriptional activity of the rice genome as detected by tiling arrays.

### Non-exonic TARs are conserved between *japonica* and *indica* rice

Comparison of the non-exonic TARs between the two subspecies showed that 8515 *japonica* TARs overlap with 7939 TARs from *indica*. In addition, 1713 *japonica* non-exonic TARs overlap with 1503 *indica* gene models (Figure 1C). Thus, 10,228 (40%) *japonica* non-exonic TARs have a direct match in the *indica* transcriptome and 1713 correspond to locations that may be misannotated as non-genic sequences in the initial genome annotation.

Because TARs were identified as transcribed regions of the genome, they likely represent fragments of larger discrete transcription units. Thus, *indica* and *japonica* TARs representing different portions of the same transcription units may not overlap directly. To facilitate examination of the conservation of *indica* and *japonica* TARs, we first identified a set of gene models shared between *japonica* (8921) and *indica* (8925) and supported by full-length cDNA sequences. These common gene models presumably represent the most conserved portion of the rice transcriptome. Within our data sets there were 3449 *japonica* and 4230 *indica* exonic TARs derived from 2808 and 3461 confirmed shared gene models, respectively. We found that 1595 (46%) *japonica* TARs overlapped with 1599 (38%) *indica* TARs (Figure S3). Thus, non-exonic TARs and exonic TARs from confirmed gene models contained similar portions that exhibit direct sequence overlap between the *japonica* and *indica* transcriptomes. These results indicate that the non-exonic TARs represent transcripts that were conserved between the two rice subspecies at a level comparable to genes.

### Transcriptional verification of non-exonic TARs

**Figure 2:**
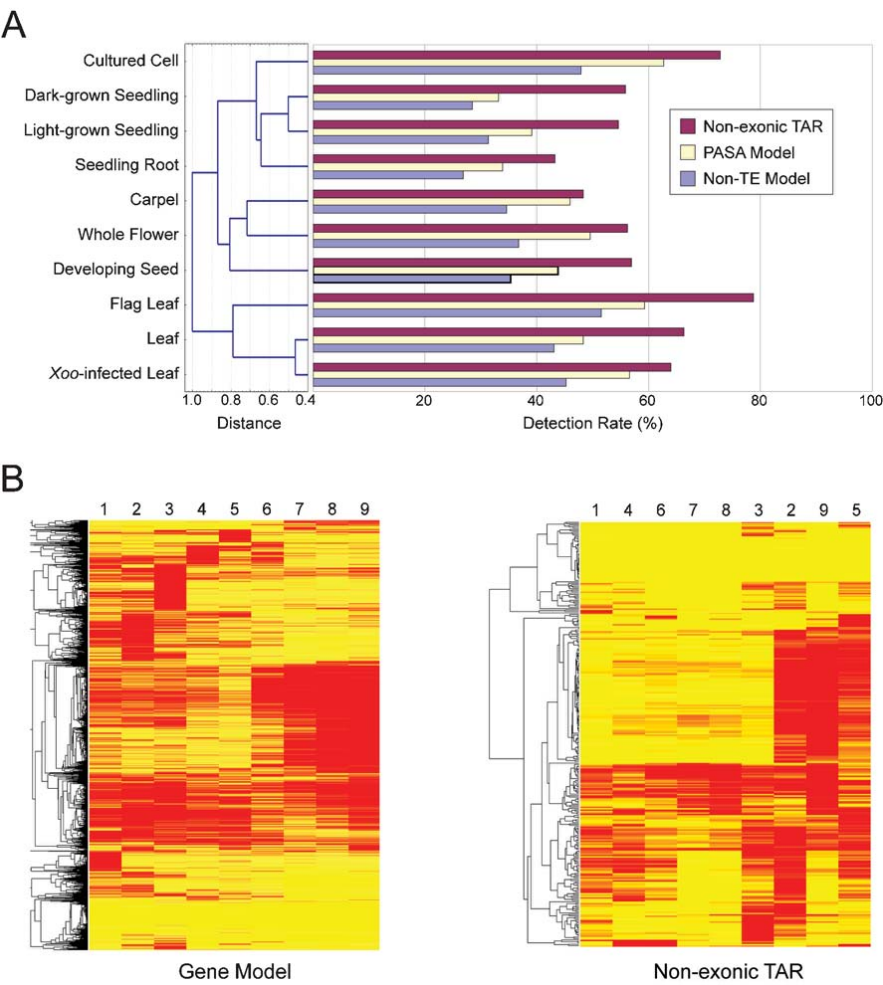
Re-array analysis of *japonica* gene models and non-exonic TARs. (A) Transcription of gene models and TARs detected by the re-array. Shown on the left is a tree diagram of the overall transcriptional relation of gene models among the 10 assayed tissue types computed with the Manhattan distance function. Expression rates in percentage of all gene models, the PASA gene models, and non-exonic TARs are shown on the right. (B) Heat map of differentially expressed gene models and non-exonic TARs. The red and yellow colors represent up- and down-regulated transcription units, respectively. The gene tree is shown on the left. Numbers on top represent the tissue types: 1, developing seed, 2, carpel, 3, whole flower, 4, dark-grown seedling, 5, seedling root, 6, light-grown seedling, 7, flag leaf, 8, leaf, 9, *Xoo*-infected leaf.

Rice TARs were scored using a method based on the binomial theorem in which at least five consecutive probes whose intensities lie in the 80^th^ percentile were identified [24]. In theory, this method should not produce false positives in excess of 10% among the 39,000 TARs from 12 million probes (12×10^6^×0.2^5^/39,000). To experimentally validate transcription of the non-exonic TARs and to describe their transcriptional regulation, we constructed a new array (designated the re-array) using five independent 36-mer probes to represent each of the 44,385 non-TE gene models and 25,313 TARs in the *japonica* genome (see Materials and Methods). Using the re-array, we measured expression in triplicate for 10 different rice organs (Figure 2A and S4). Transcription of 7965 (18%) and 28,265 (64%) gene models were detected in every organ and in at least one of the assayed organ types, respectively. Among the 10 organs, the rate of gene expression detection ranged from 27% (seedling root) to 52% (flag leaf) (Figure 2A). Furthermore, the relative overall similarity of the transcription levels of gene models was consistent with the developmental and/or physiological state of the organs (Figure 2A).

PASA is a genome annotation tool that exploits spliced alignments of expressed transcripts to model gene structures [27]. PASA gene models are therefore supported by and consistent with available experimental evidence. In the analysis of the re-array 14,740 rice PASA models were represented [8], [9]. Higher detection rates for the PASA models, than for the 44,385 genes in the genome annotation, were obtained for all 10 organ types, which ranged from 33% (seedling grown in dark) to 63% (cultured cell), and 11,542 (78%) of the PASA gene models were detected in at least one organ type (Figure 2A). These results indicate that the re-array was an efficient method to detect expression from known rice transcripts.

From the re-array, transcription of 9323 (37%) and 21,018 (83%) non-exonic TARs was detected in all and at least one organ types, respectively. Across organ types, detection rates of TARs ranged from 43% (seedling root) to 79% (flag leaf) (Figure 2A). We further observed that re-array detection rate of TARs was consistent with other experimental evidence. First, there were 3473 non-exonic TARs that had sequence matches to cDNA sequences among the rice Unigenes (see Materials and Methods) (Figure 1A). Similar to the detection of exon-derived TAR, Unigene-supported non-exonic TARs exhibited a detection rate of 90% while 82% those without a Unigene match were confirmed in the re-array (Figure S5). Second, we performed reverse transcription coupled PCR (RT-PCR) analysis on 108 randomly selected non-exonic TARs. The Unigene-supported and re-array detected TARs exhibited higher PCR confirmation rates (92% and 78% respectively) than that (44%) of TARs not detected by the re-array (Figure S5).

We determined which genes and non-exonic TARs that were differentially expressed among the 10 assayed organ types. While 4232 (9.5%) genes were differentially expressed between organ types, fewer (545, or 2.2%) non-exonic TARs than genes were differentially expressed across the organ types assayed (Figure 2B). Together, our results indicate that non-exonic TARs are generally more ubiquitously transcribed than genes. Interestingly, detection rates of non-exonic TARs were highly correlated with the gene detection rates (Pearson correlation coefficient *r* = 0.92), suggesting that the transcriptional activity represented by non-exonic TARs reflects the activity of the transcriptome and is thus a worthy target for future transcriptome profiling efforts.

### Non-exonic TARs correspond to portions of existing gene models

**Figure 3:**
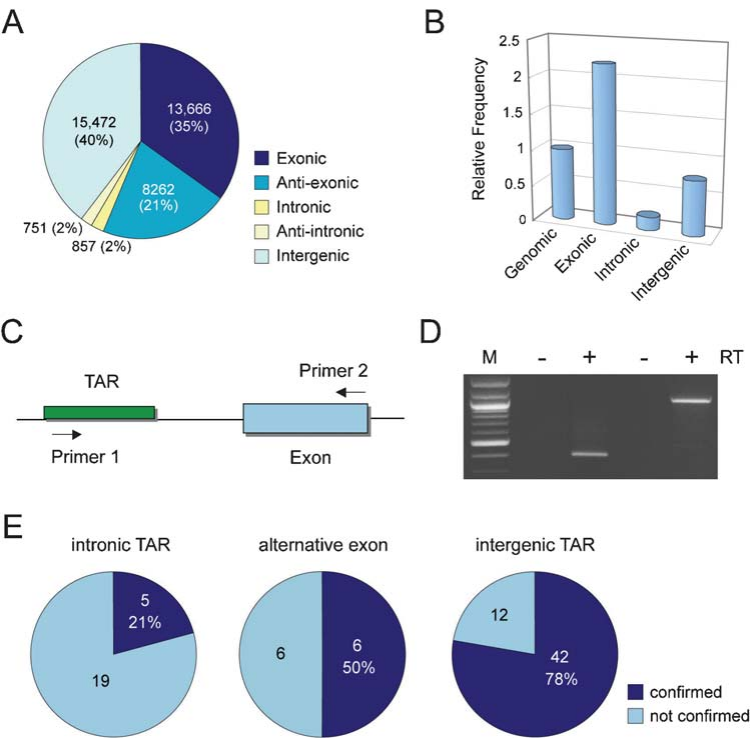
Analysis of non-exonic TARs representing portions of annotated gene models. (A) Number of TARs in different components of the rice genome. (B) Relative frequency of TARs in different genomic compartments. Note that strandedness of the TARs was not considered in this analysis. TAR frequency was calculated as the number of TARs per Mb and normalized to the genomic TAR frequency. (C) The RT-PCR assay to examine whether a gene/TAR pair belongs to the same transcript in which primer pairs target the gene model and the TAR in a convergent fashion. (D) Examples of PCR products amplified in a reverse transcription (RT) dependent manner for the gene/TAR pairs Os10g21310 and Chr10fwd_10366435 (left), and Os02g50070 and Chr2fwd_30528038 (right). (E) Confirmation rates obtained for intronic TARs and proximal intergenic TARs by RT-PCR.

**Figure 4:**
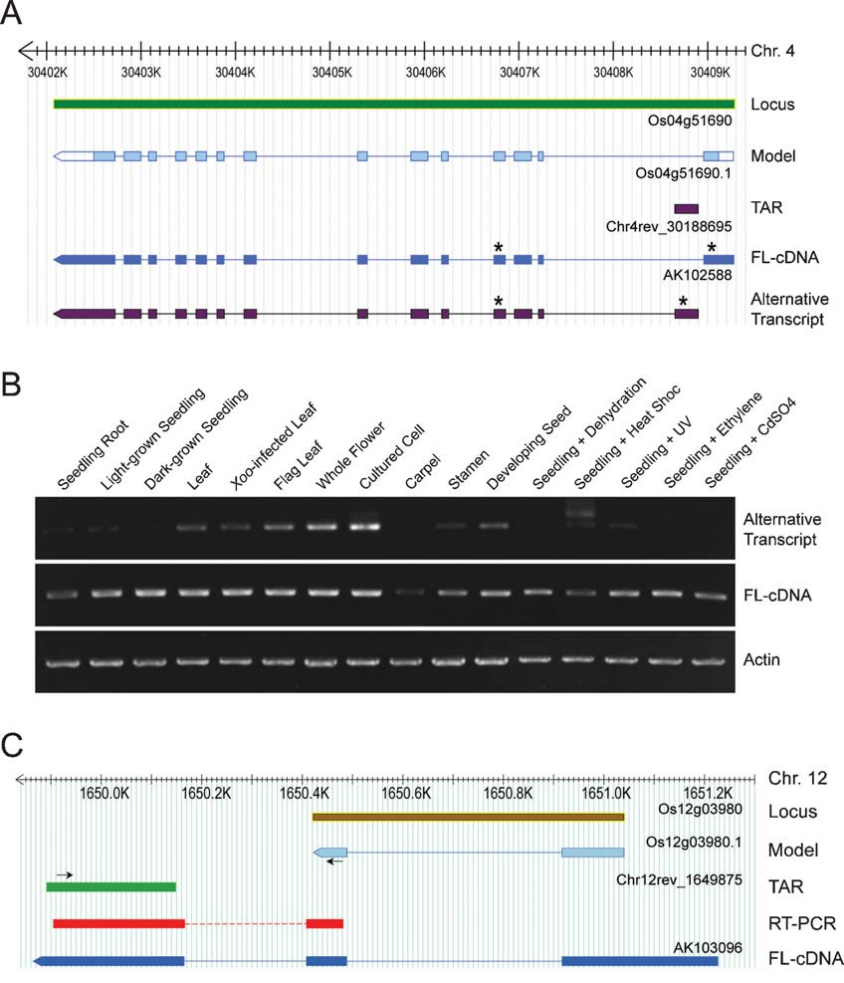
Experimental verification of selected non-exonic TARs representing uncharacterized portions of gene models. (A) Annotation of an intronic TAR. The TAR Chr4rev_30188695 is in the first intron of the gene model Os04g51690. A TAR-containing transcript was proposed in which the TAR serves as the alternative first exon. (B) RT-PCR analysis of the transcript abundance in diverse conditions for the FL-cDNA AK102588 and the TAR-initiated alternative transcript. Positions of the primers are indicated by the asterisks shown in (A). (C) Annotation of a proximal intergenic TAR 3′ to a gene model. Arrows indicate the primer positions.

Introns of the rice genome were relatively depleted of transcripts represented in the form of TARs (Figure 3A and 3B). This is because only intronic TARs present in a poly(A)-baring transcript would have been detected under our experimental conditions. We used an RT-PCR assay in which a pair of primers was designed to target the intronic TAR and a downstream exon to examine whether the intronic TARs could represent splice variants of the related genes (Figure 3C). However, only five out of 24 selected cases (21%) were unambiguously verified (Figure 3D and 3E). We observed that the frequency of intronic TARs correlated with the median sizes of the introns (*r* = 0.97). This prompted us to investigate whether TARs in the first introns, which are the largest and have the most TARs (358) among all introns, could represent alternative first exons that have been reported to contribute substantially to transcript diversity in mammals but only sparsely documented in plants [28]. Through RT-PCR analysis, six of the 12 (50%) randomly selected cases were confirmed in which the intronic TAR was found as part of an alternative transcript derived from the annotated locus (Figure 3E). Detailed annotation of one such example is illustrated in Figure 4A. The novel TAR-initiated splice variant and the annotated gene model exhibited distinct transcription pattern across the examined conditions (Figure 4B), indicating that the alternative transcripts were differentially expressed.

There were 15,472 TARs in the intergenic regions (Figure 3A) of which 1682 were located in close proximity (<1 kb) and on the same strand as a gene model. In addition, 523 TARs exhibited strong co-transcription pattern (*r*>0.7) with the corresponding gene model. As many of the rice gene models were only annotated for their coding regions [8], we speculated that the proximal intergenic TARs could correspond to untranslated regions or misannotated coding capacities of the nearby gene models. To test this, we performed RT-PCR analysis using primer pairs to test for physical linkage of 54 randomly selected TARs to proximal gene models. In this sample 42 (78%) generated a PCR band in an RT-dependent manner, indicating that the co-transcribed TARs represent previously uncharacterized portions of annotated gene models (Figure 3E and 4C). Assuming the PCR confirmation rates can be applied to all non-exonic TARs within or close to annotated gene loci (857×21%+358×50%+523×78%), these results indicate that ∼760 (3%) TARs represent various un-annotated portions of the existing gene models (Table 1).

**Table 1:** Summary of characterization of non-exonic TARs in *japonica* rice.

TAR Location	Number	Putative Functional or Structural Elements	Extrapolated Number
Intronic	857 (3.4%)	Alternative first exons	179 (0.7%)
		Other splice variants	180 (0.7%)
Antisense Intronic	761 (3.0%)	Antisense transcripts	761 (3.0%)
Antisense Exonic	8262 (32.6%)	Expressed antisense transcripts	7061 (27.9%)
		Negatively correlated with gene models^2^	884 (3.5%)^3^
		Non-coding transcripts	3120 (12.3%)^3^
Intergenic	15,472 (61.0%)	Un-reported distal portion of gene models	408 (1.6%)
		Pack-MuLE related	1850 (7.3%)
		Non-coding transcripts	10,500 (41.4%)
Total	25,352 (100%)^1^		20,939 (82.6%)

Transcription of the non-exonic TARs was determined by the re-array. The non-exonic TARs were linked to various putative functional or structural elements of the rice genome based on current annotations. Number of non-exonic TARs corresponding to each group of functional elements was extrapolated to the full genome based on experimental confirmation rates where applicable and the physical sizes of the annotated regions relative to that of the genome.

1 This number excludes 13,666 TARs intersecting with annotated exons.

2 Transcription levels exhibiting a Pearson correlation coefficient <−0.4;

3 Redundant with expressed antisense transcripts and not included in the total.

### Identification of non-exonic TARs antisense to annotated gene models

**Figure 5:**
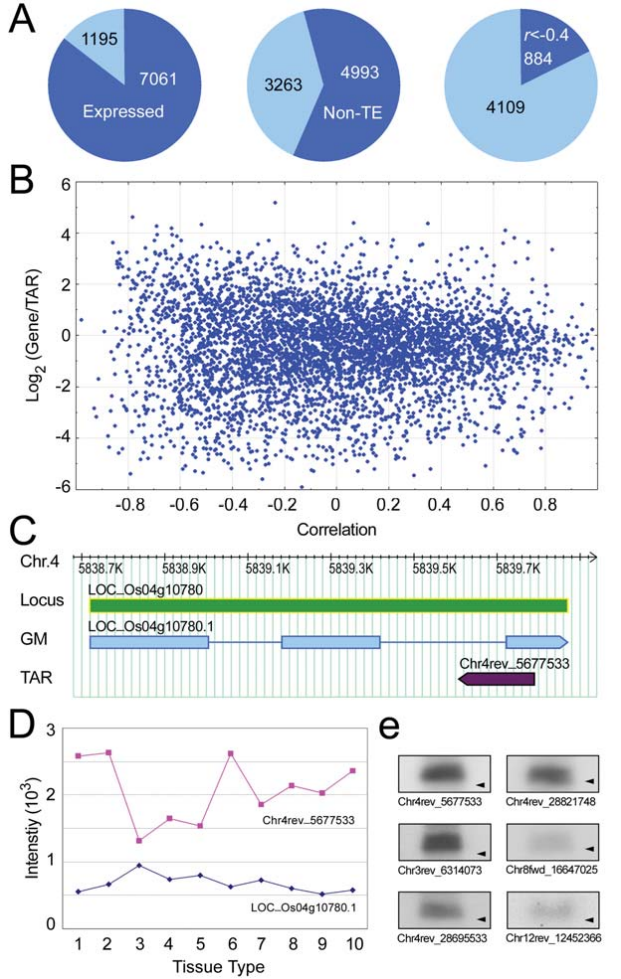
Characterization of antisense TARs. (A) Pie chart analysis of antisense TARs. Of the 8256 antisense TARs, 7061 were expressed in at least one tissue type according to the re-array (left), 4993 were antisense to a non-TE gene model (center), of which 884 had a transcriptional correlation coefficient with the corresponding gene models in the 10 assayed tissue types <−0.4 (right). (B) Scatterplot of the relative transcription level of the gene model/antisense TAR pairs in the cultured cell versus their correlation coefficient across the 10 assayed tissue types. (C) Annotation of an antisense TAR. The TAR Chr4rev_5677533 overlaps convergently with the gene model Os04g10780. (D) Transcription level of the TAR Chr4rev_5677533 and the gene model Os04g10780 in the 10 assayed tissue types. 1, cultured cell, 2, seedling root, 3, dark-grown seedling, 4, light-grown seedling, 5, leaf, 6, *Xoo*-infected leaf, 7, flag leaf, 8, whole flower, 9, carpel, and 10, developing seed. (E) Northern blot analysis of small RNA related to antisense TARs. The probes were PCR products derived from antisense TARs. The migration positions of a 21 nt RNA are indicated by the black triangle.

Natural antisense transcripts (NATs) have been broadly implicated in regulating gene expression and chromosome structure [29]–[34]. Large-scale efforts to systemically identify NATs have been made in several model organisms [35]–[37]. In rice, 687 bi-directional transcript pairs were identified from a collection of 32,127 FL-cDNAs [38]. Analysis of our tiling array data detected 9023 non-exonic TARs located on the opposite DNA strand of annotated gene models that potentially represent *cis*-NATs. Antisense TARs exhibited slightly higher re-array detection rate than TARs on average. For example, 8256 of 8262 anti-exonic TARs were represented in the re-array of which 7061 (85%) were detected (Figure 5A). These observations suggest that rice NATs may be more prevalent than previously realized.

A total of 4993 TARs antisense to annotated exons in the non-TE genes were represented on the re-array, allowing us to investigate the transcriptional pattern of the TAR/gene pairs across the examined tissue types. The transcription of 884 antisense TAR/gene pairs showed strong negative correlations (*r*<−0.4) (Figure 5A). This group of antisense TAR/gene pairs also had greater difference in their transcription level than other pairs (Figure 5B), manifesting a strong reciprocal transcription pattern in the assayed tissue types.

Because antisense transcripts can function through small interfering RNA (siRNA) derived from paired sense–antisense transcripts [29]–[31], we investigated whether the negatively correlated antisense gene/TAR pairs could generate siRNA by Northern blot analysis using the cloned TARs as probes (Figure 5C–5E). This analysis detected low-molecular-weight RNA bands of similar size to known siRNA in six out eight cases (Figure 5E). Detailed analysis of one antisense TAR is illustrated in Figure 5C–5E.

### Analysis of TARs intersecting Pack-MuLEs

We examined the intersection of TARs with elements of the genome other than the annotated protein-coding genes. Chimeric *Mutator*-like transposable elements carrying fragments of genes, so called Pack-MuLEs, are a characteristic of plant genomes and may be involved in the creation of new genes during evolution [39], [40]. In rice, 475 Pack-MuLEs in Chromosome 1 and 10 were previously annotated in detail [39]. We report here the analysis of TARs associated with these annotated Pack-MuLEs in Chromosome 1 and 10. We observed that 248 Pack-MuLEs match with at least one of the TARs (446 in total, including 118 exonic and 328 non-exonic TARs), indicating that about half (248/475 = 52%) of the Pack-MuLEs were potentially transcribed. Extrapolating our observations on Chromosome 1 and 10 to the whole genome (328×390Mb/69Mb) suggests that as many as 1850 (7.3%) non-exonic TARs could be encoded by Pack-MuLEs (Table 1).

We found that 109 TARs intersected 96 ORFs encoded by Pack-MuLEs whilst 112 were located on the antisense strand of 97 ORFs. Among these, 48 ORFs were intersected by both sense and antisense TARs. Such a strong symmetric transcription pattern of Pack-MuLEs consisting of equal number of sense and antisense TARs is in keeping with the observation that most Pack-MuLE derived FL-cDNA were initiated within or near the terminal inverted repeats (TIRs) [39]. In further support of this conclusion, we found 63 TARs with significant match to the TIR sequences (E≤10^−10^). Although it is possible that the detected transcriptional activity of Pack-MuLEs were derived from close homologs elsewhere in the genome, the strong symmetric transcription pattern argues against this possibility. The symmetrical pattern of transcription suggests that Pack-MuLE loci could be a rich source for *trans*-regulatory RNAs (i.e. double-stranded or *trans*-antisense) that can potentially affect expression of the original genes [29]–[34], [41].

### Intergenic TARs and non-coding transcripts

**Figure 6:**
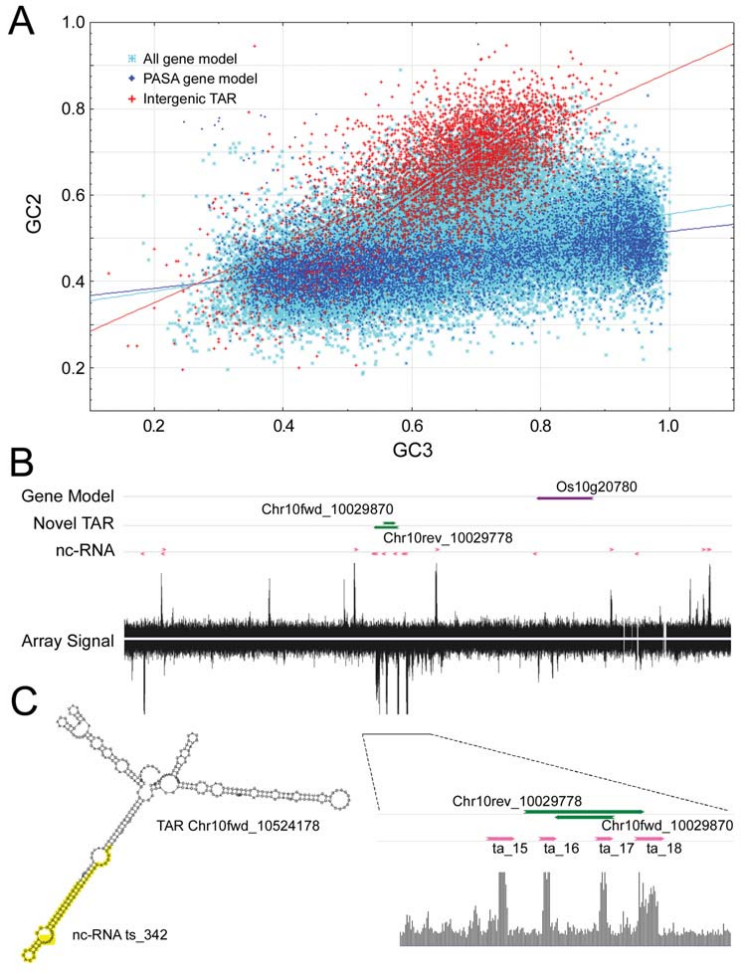
Analysis of non-coding intergenic TARs. (A) Scatterplot of GC2 versus GC3 in all gene models (n = 46,976), FL-cDNA-supported PASA gene models (n = 11,494), and intergenic TARs (n = 5256). The intergenic TARs were distal (>1Kb) to a gene model excluding those with a hit in the ProSite database. (B) Overlapping TARs with putative non-coding transcripts. A 5-Kb region represented by the high-resolution tiling array is shown. The interrogating probes are aligned to the chromosomal coordinates, with the fluorescence intensity value depicted as a vertical line. Gene models, no-exonic TARs and putative non-coding transcripts are depicted as horizontal arrows, which point to the direction of transcription. A portion of the region containing four non-coding transcripts and a pair of TARs is enlarged and shown at the bottom. (C) Predicted secondary structure of the TAR Chr10fwd_10524178. The sequence corresponding to the putative nc-RNA ts_342 is highlighted.

The intergenic TARs, distal to a gene model and not overlapping with other elements of the genome, numbered ∼8400 in total and do not appear to encode proteins. Two lines of evidence supported conclusion. First, the linear relationship between the GC content of the second (GC2) and the third (GC3) codon positions of the longest deduced ORF, of the six possible translations, from these TARs deviated from that of known genes [42]. As shown in Figure 6A, the GC3/GC2 correlation of the intergenic TARs aligned along the diagonal, far from that of the PASA gene models. Consistent with this observation, the TAR-deduced peptide sequences were enriched for GC-rich codons, such as Pro and Arg (Figure S6). Although it is possible that some of the putative proteins have legitimate biological functions, the biased codon usage suggests that many of the intergenic TARs may not function through their deduced ORFs.

The non-exonic TARs were compared to putative non-coding RNA (nc-RNA) identified using a high resolution (5-bp) tiling array. This array, which represents a 1Mb region of Chromosome 10, was hybridized to mRNA- or small RNA-derived cDNA samples. Hybridization signals from labeled mRNA-derived cDNA matched known transcripts with high fidelity (Figure S7). When this array was hybridized to cDNA derived from specifically enriched small nc-RNA samples [43], 27 of the 29 annotated tRNA genes in this region were detected, indicating that tRNA-sized nc-RNAs were well represented in the labeled sample. In addition, 47 of the hybridization signals corresponding to nc-RNAs overlapped with 35 non-exonic TARs. Eight of the TARs were antisense to exon sequences and intersected 12 nc-RNAs while 27 intergenic TAR intersected 35 nc-RNAs within the 1Mb region interrogated by the array. Extrapolating these results to the whole genome suggests that there could be ∼10,500 (27×390Mb/1Mb) intergenic TARs and ∼3120 (8×390Mb/1Mb) antisense TARs related to non-coding small RNA species (Table 1). Interestingly, six intergenic TARs, which intersected 13 nc-RNAs, also have their own antisense TARs (Figure 6B), suggesting that the small RNA species might be derived from the paired antisense transcripts.

**Figure 7:**
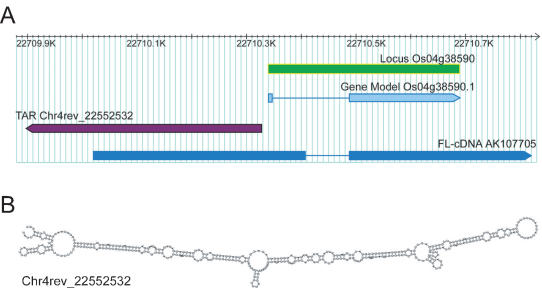
Analysis of 5′ proximal antisense TARs. (A) Annotation of TAR Chr4rev_22552532 that locates in a divergent antisense orientation to the gene model Os04g38590. (B) Predicted secondary structure of the RNA transcript derived from the TAR Chr4rev_22552532. RNA secondary structure was predicted using RNAfold, which is available as part of the Vienna RNA Package.

Some TARs with corresponding small RNA derivatives were predicted to fold into stable secondary structures in which the nc-RNAs were located near stem-loop motifs (Figure 6C). It is a possibility that the small nc-RNAs are processed from these corresponding TARs. We were also able to predict stable secondary structures for the many intergenic TARs not represented by the 1Mb tiling array, particularly those located in close proximity to gene models in a divergent antisense orientation (Figure 7). We identified 234 intergenic TARs that have homology to sequences in different plant species. These clusters of sequence homologs were used to search for structural conservation using the RNAz algorithm [44], which uses comparative sequence analysis and RNA secondary structure prediction to detect evolutionarily conserved and exceptionally stable secondary structures. By this method, 32 of these 234 non-coding TAR clusters (14%) were predicted form stable secondary structures. This fraction is significantly higher (binomial *p*<*e* ^−15^) than that for randomly shuffled TAR alignment blocks, for which only ∼6% are predicted to form structural nc-RNA.

In summary, we identified 25,352 non-exonic TARs in the *japonica* genome that represent conserved transcripts between *japonica* and *indica* rice. We verified transcription for 83% of these TARs through expression profiling and assigned more than 80% TARs to various putative functional or structural elements of the rice genome (Table 1). The putative novel transcripts tagged by non-exonic TARs should therefore be useful to improve rice genome annotation and facilitate comparative genomics in other related but more complex cereal genomes. Among the non-exonic TARs, 3473 matched with the current rice Unigene set (Figure 1A), suggesting that those transcripts were missed from the genome annotation we used. In addition to the novel potentially protein-coding transcripts, many of the non-exonic TARs do not appear to encode proteins. Many of these non-exonic TARs were arranged into or transcribed from specific configurations with protein-coding genes, such as duplicated gene fragments or antisense transcripts (Table 1), and may function to modulate the genes' expression. The non-exonic TARs that could not be directly linked to annotated genes were often associated with small non-coding RNA species (Figure 6). These results thus expand the current view of the complexity and dynamics of the rice transcriptome.

## Materials and Methods

### Plant material and RNA preparation

*Oryza*. *sativa* ssp. *japonica* cv. Nipponbare was used for all experiments. Light- and dark-grown seedlings, suspension cultured cells, panicles, and stress-treated seedlings were prepared as previously described [14]. For other materials, seedlings were transferred to soil and maintained under long-day conditions (16 hour light/8 hour dark) at 26–28°C in the greenhouse. After 7 weeks, mature leaves were inoculated with the pathogen *Xanthomonas oryzae* pv. *oryzae* race PXO99 (*Xoo*) using the leaf clip method [45]. During the flowering stage, flag leaves, flowers (florets), stamens and carpel (before pollination) were harvested. Developing seeds were harvested three to four days after pollination. For microarrays, total RNA and mRNA were sequentially isolated using the RNeasy Plant Mini kit (Qiagen) and the Oligotex mRNA kit (Qiagen). For Northern blot analysis, total RNA was isolated from rice seedlings using the Trizol method (Invitrogen). Low-molecular-weight RNA was enriched and subsequent Northern blot analysis carried out as previously described [33]. Non-coding RNA species were isolated from total RNA as previously described [43]. Fractions corresponding to tRNAs (sample I) and small RNAs of 80–500 nt (sample II) were collected separately.

### Microarray design

Three different types of microarrays were used in this study: the full-genome tiling arrays, a 1Mb high-resolution tiling array, and a re-array. All arrays were fabricated at a density of 389,000 oligonucleotides per array as previously described [14], [15], [24]. For the full-genome tiling arrays, we designed a minimal tiling path employing 36-mer oligonucleotides spaced by 10 nt on average to represent the *japonica* rice genome, based on the TIGR Rice Pseudomolecules release 1 (September 2003) [8]. Details regarding design of the genome tiling array can be found in a previous study [15]. The 1 Mb tiling array was used to represent the 10.0 Mb to 11.0 Mb region of *japonica* rice Chromosome 10. A tile path was designed with 36-mer probes at a step of 5 nt.

We constructed the re-array in which five probes were used to represent each gene model and non-exonic TAR. To ensure that the probes are specific to its target and are free of secondary structures, we used the software OligoArray 2.1 to select probes [46]. We started with 44,497 gene models and 25,352 non-exonic TARs, which were used as the database sequence to search against. Two separate runs were initiated, one for the gene models and the other for the TARs. For transcription units for which the software could not generate five probes, the remaining probes were selected from the original tiling arrays that were in the 75^th^ percentile and met most of the above criteria (hence the name ‘re-array’). 10,000 randomly selected probes from the genome tiling arrays and 9000 probes in the introns of PASA models with intensities below the median were also included as controls.

The full-genome tiling arrays and the 1Mb tiling array were hybridized to cDNA mixtures derived from seedling root, seedling shoot, panicle and suspension-cultured cells. Note that the cDNA mixture was used as the template for RT-PCR analysis unless otherwise indicated. Additionally, the 1Mb tiling array was hybridized to targets derived from the non-coding RNA Sample I and II. The re-array was hybridized to three biological replicates of cDNA derived from 10 rice tissue types, namely, light-grown seedling, dark-grown seedling, seedling root, leaf, *Xoo*-infected leaf, flag leaf, whole flower (floret), carpel, developing seed, and suspension cultured cell.

### Full-genome tiling array scoring and TAR identification

We were able to map >95% of the 12,254,374 probes to the latest TIGR Rice Pseudomolecules (release 3, release date 02.18.05) at the time data analysis was initiated. We used the gene model annotation provided in the form of a .gff file distributed by TIGR [8], from which we identified 62,121 gene models (mRNA transcripts), including 13,467 that were TE-related. We excluded gene models that were represented by less than five probes, as one cannot do statistical tests on them. We also excluded 11,143 gene models that were not sufficiently probed, i.e., <45% coverage of their coding region by tiling probes. There were 2549 loci that generated 5897 alternative gene models of which only one from each locus was included. Thus, a total of 5719 TE and 40,257 non-TE models were represented on the tiling arrays.

We used the sign-test to score annotated gene models [24]. We first identified probes that lie within the exons of a given gene model. For each probe, we checked if its intensity was greater than the median of the array to which it belongs. For each gene model, we determined whether or not the number of probes that are above the median is more than expected by chance alone. The probability, p, of obtaining h probes with intensities above median out of N probes is given by the equation:
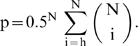
1We chose a p-value cutoff of 0.05 (corresponding to a false-positive rate of 5%), meaning that gene models with a p-value less than 0.05 were scored as transcribed [24], [47].

To identify TARs, the tile path was scanned for regions in the genome that are covered by at least five consecutive probes whose intensities lie in the 80^th^ percentile [24], [47]. To accommodate for shifted and overlapping probes because of re-mapping, we further applied a minimal length cutoff of 220 nt and a gap penalty of 30 nt between two probes. Using these criteria, we identified a total of 39,018 and 42, 470 TARs from the *japonica* and *indica* genome, respectively. To match non-exonic TARs to rice Unigenes (sets of transcript sequences derived from the same transcription unit), which were downloaded from NCBI (http://www.ncbi.nlm.nih.gov/UniGene/UGOrg.cgi?TAXID = 4530), a cutoff of E≤e^−10^ was used.

### Re-array scoring

We developed the NMPP package as a bundle of user-customized tools based on established algorithms and methods to process the re-array data [48]. The package is available at: http://plantgenomics.biology.yale.edu/nmpp. To correct potential skewed signal distribution within each array due to uneven hybridization or washing, we applied a global distance-weighted spatial smoothing to the raw probe intensities based on *MAS* 5.0 [48]. We follow a two-step process to normalize the smoothed intensity values. First, we performed a quantile normalization of the three replicates within each tissue type. We next carried out a global scaling to adjust the quantile-normalized intensity to the same baseline, which is the median of all the intensity values. For each transcription unit, we obtained a 5×3 matrix consisting of the normalized intensity values from five probes in triplicate. We assigned a value to a transcription unit based on this matrix to represent its transcription level using the Tukey's median polish procedure [48].

Of the 19,000 control probes included in the re-array, 7669 have a GC content higher than 15, which is the case for all probes interrogating transcription units. These 7669 probes were used as the control for subsequent analysis of the re-array data to minimize the impact of probe GC content on transcription calling. To determine from the control probes the noise distribution, we used a previously reported modeling process [14]. Assuming that control probes with very low intensity (1-9 Log_2_, Supplementary Fig. 4) were primarily noise, we modeled the noise as a normal distribution by mirroring the low-intensity portion of the control probe distribution. For transcription detection of gene models or TARs, the calculated transcription level of each unit was compared with the noise distribution. We used a false-discovery rate (FDR) of 0.05, which represents the 95% confidence in the modeled noise distribution, as a cutoff for determining whether the transcriptional intensity of a given transcription unit is higher than the noise.

Determination of differentially expressed transcription units was carried out using standard procedures. Briefly, normalized intensity values were fitted to a mixed effects ANOVA model (R/maanova, version 1.2.1, http://www.jax.org/staff/churchill/labsite/software/download.html). An *F*-test was performed to detect differential expression of gene models or non-exonic TARs in the 10 tissue types. Next, FDR-adjusted *p* values were calculated from permuted samples. A significant level above 99% (*p*<0.01) was selected for tissue-specific expression calling of gene models or TARs.

### 1Mb tiling array scoring

We converted probe intensity values from the 1 Mb tiling array into Z scores using a two-step process. First, the mean (μ_G_) and the standard deviation (σ_G_) of all intensity values were calculated and probes with intensity greater than μ_G_+2σ_G_ were marked as group I probes. Next, a new mean (μ_G′_) and a new standard deviation (σ_G′_) were calculated excluding the group I probes. The Z scores for the *i* ^th^ probe is given by the equation z_i_ = (x_i_-μ_G′_)/σ_G′_. Probes with a Z score greater than two were considered signal probes. Sequences of neighboring signal probes were joined to form contigs. Putative non-coding transcripts were identified from the contigs with a minimal length of 55 nt (sample I) or 65 nt (sample II).

### RNA secondary structure analysis

RNA secondary structures were predicted using RNAfold. To verify the secondary structures, we used 15,000 TARs sequences to BLAST search five plant Unigene databases (maize, wheat, barley, sorghum and Arabidopsis) downloaded from NCBI. This search identified 708 hits (E<e^−10^, identity ≥ 70%, and minimum alignment length >50 bp). Clusters of TARs and sequence homologs were aligned using the CLUSTALW algorithm to generate multiple sequence alignment blocks suited for RNAz analysis [44]. Alignment blocks of a minimum length of 40 were subjected to RNAz, utilizing an offset of 40 (for alignment blocks >80) and considering both DNA strands independently. Regions with an RNAz support vector machine (SVM) classification score P>0.5 were collected. As a control, multiple sequence alignment blocks were shuffled using a randomization algorithm that takes care not to introduce randomization artifacts and produces random alignments of the same length, base composition, overall conservation, local conservation pattern, and gap pattern as the input alignment [44].

### Data availability

Information on *japonica* rice TARs can be viewed and retrieved at this website: http://dart.gersteinlab.org/rice/. Microarray data for the full-genome tiling array, the 1Mb tiling array, and the re-array are available in the NCBI Gene Expression Omnibus under series GSE6996, GSE6921, and GSE6922, respectively.

## Supporting Information

Figure S1 Tiling array detection of annotated gene models in rice. The tiling arrays were hybridized to a mixture of cDNA targets derived from four major rice tissues to optimize transcript representation (see Methods). The 62,121 annotated rice gene models include 13,467 TE and 48,654 non-TE models. Of these, 40,257 non-TE models (a), 14,285 PASA models (a set of improved gene models from incorporation of EST and FL-cDNA information) (b), and 5719 TE models (c) were considered uniquely and sufficiently represented in the tiling arrays. While 21,391 (53%) non-TE models were detected, a higher detection rate (73%) was obtained for PASA models, attesting to the accuracy of tiling array detection. Of the 5719 TE models, 1716 (30%) were detected, indicating that a significant portion of the rice TE models is actively transcribed.
(0.24 MB PDF)

Figure S2 Density of different sets of transcription units along the 12 rice chromosomes. The number of transcription units was calculated in 500 Kb-sliding-windows with a 100 Kb step. The black triangle sign in each panel indicates the position of the annotated centromere of the corresponding chromosome.
(1.29 MB PDF)

Figure S3 Comparative analysis of TARs. (a), Comparison of 5464 indica TARs with transcription units in japonica. The indica TARs were identified in a previous genome tiling analysis of the indica genome (Ref.2). Exon, indica TARs matched with exons of japonica genes, Antisense Exon, indica TARs mapped to the antisense strand of japonica genes, TAR, indica TARs matched with japonica novel TARs, Antisense TAR, indica TARs mapped to the antisense strand of japonica novel TARs, Unmatched, indica TARs do not match with any japonica transcription unit, Unmapped, indica TARs not mapped to the japonica genome. (b), Classification of 42,470 indica TARs based on their physical relation with annotated indica gene models. The 42,470 TARs were identified by re-analyzing the previous indica genome tiling data (Ref.2) using the same criteria described in Methods. (c), Intersection of exonic TARs with common FL-cDNA supported gene (CG) models. Gene models in both japonica and indica were aligned to rice FL-cDNA to identify the CG models, which were mapped against each other using BLAT. Models were considered common if they overlap >100 bp in the annotated ORFs (Ref.1). Exonic TARs from japonica (green) and indica (red) were matched against the common CG models (blue) and represented by the cyan and magenta color, respectively. TARs matched to the CG models in japonica and indica were compared using BLAT and considered common if they overlap by at least 1 bp (white).
(0.25 MB PDF)

Figure S4 Re-array data processing. (a) Normalization of the re-array hybridization data. Spatial-effect smoothed probe intensities were normalized following a two-step process. First, quantile normalization was performed on the three replicates within each assayed tissue type. Next, a global scaling was carried out to adjust the quantile-normalized intensities to a common baseline. (b) Modeling the 7669 control probes. To determine from the control probes the noise distribution, we used a previously reported modeling process (Ref.1). Assuming the control probes that have very low intensities (1-9 Log2) were primarily noise, we modeled the noise as a normal distribution by mirroring the low-intensity portion of the overall control probe distribution. (c) Transcription threshold determination for individual tissue types. FDR = 0.05, which represents the 95% confidence in the modeled noise distribution, was used as a cutoff for transcription calling.
(1.25 MB PDF)

Figure S5 Transcriptional verification of non-exonic TARs. (a) Re-array detection of different groups of TARs. Detection rate was calculated as the percentage of TARs detected in at least one of the 10 assayed tissue types by the re-array. All, all non-exonic TARs; +Hit, TARs match with rice Unigenes; -Hit, TARs do not match with Unigenes. (b) RT-PCR analysis of selected non-exonic TARs. Pie charts indicate the percentage of TARs generated a PCR product of the expected size in an RT-dependent manner. PCR for 36 TARs from each group (108 in total) was performed on reverse transcribed cDNA (+RT) and mRNA (-RT) and resolved on gel side by side. A typical gel from each group is shown. Top, TARs detected by the re-array and have hits against Unigenes; middle, TARs detected by the re-array but have no Unigene hits; bottom, TARs not re-array detected and have no Unigene hits. M, 100 bp molecular weight marker.
(0.88 MB PDF)

Figure S6 Analysis of the coding potential of intergenic TARs. (a) Frequency of the amino acids contained in the deduced protein sequences of rice FL-cDNA, TE and non-TE gene models, and intergenic TARs. (b) Pie chart analysis of the hits against the ProSite database using deduced polypeptide sequences from the intergenic TARs.
(0.22 MB PDF)

Figure S7 High-resolution tiling array analysis of mRNA target. (a) A region from rice Chromosome 10 represented by the tiling array. (b) Tiling array profile showing each probe aligned to the chromosomal coordinate with its fluorescence intensity depicted as a vertical line. (c) TIGR working gene model from the locus LOC_Os10g21910, which encodes a putative acetyl-coenzyme A carboxylase ACC1A. (d) Gene predictions and experimental evidence supporting the gene model. Red bars indicate significant matches with the working gene model. Data downloaded from the TIGR rice genome annotation database at http://rice.tigr.org/.
(1.28 MB PDF)
