# Supplementary figures and images for: Global Identification and Characterization of Transcriptionally Active Regions in the Rice Genome
Source: PLoS One. 2007 Mar 14;2(3):e294. doi: 10.1371/journal.pone.0000294 (PMC1808428; doi:10.1371/journal.pone.0000294)

A

Non TE Model

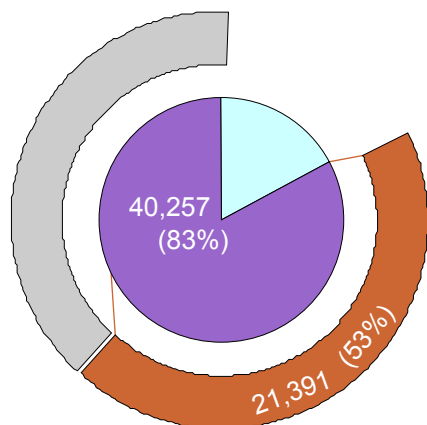

B

PASA Model

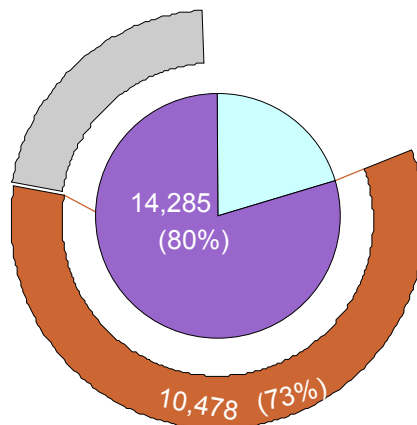

C

TE Model

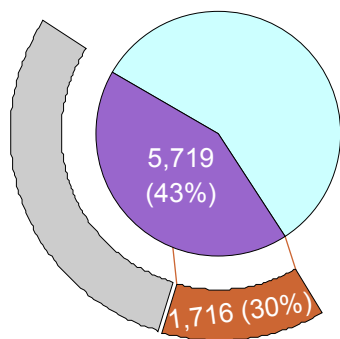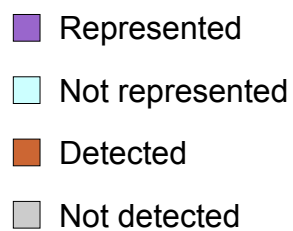

Supplement: Figure S1 — Tiling array detection of annotated gene models in rice. The tiling arrays were hybridized to a mixture of cDNA targets derived from four major rice tissues to optimize transcript representation (see Methods). The 62,121 annotated rice gene models include 13,467 TE and 48,654 non-TE models. Of these, 40,257 non-TE models (a), 14,285 PASA models (a set of improved gene models from incorporation of EST and FL-cDNA information) (b), and 5719 TE models (c) were considered uniquely and sufficiently represented in the tiling arrays. While 21,391 (53%) non-TE models were detected, a higher detection rate (73%) was obtained for PASA models, attesting to the accuracy of tiling array detection. Of the 5719 TE models, 1716 (30%) were detected, indicating that a significant portion of the rice TE models is actively transcribed. (0.24 MB PDF) [file pone.0000294.s001.pdf]

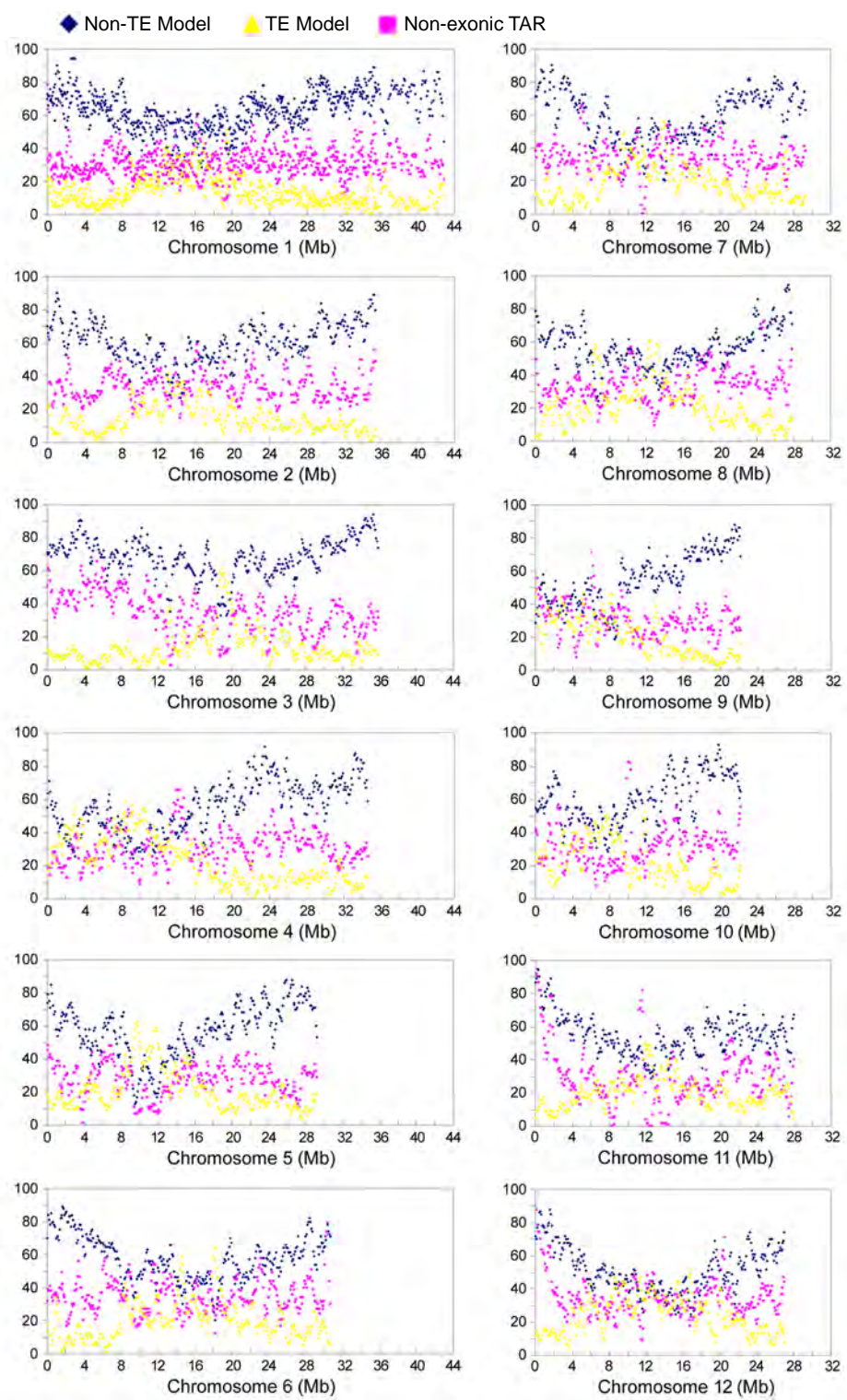

Supplement: Figure S2 — Density of different sets of transcription units along the 12 rice chromosomes. The number of transcription units was calculated in 500 Kb-sliding-windows with a 100 Kb step. The black triangle sign in each panel indicates the position of the annotated centromere of the corresponding chromosome. (1.29 MB PDF) [file pone.0000294.s002.pdf]

A

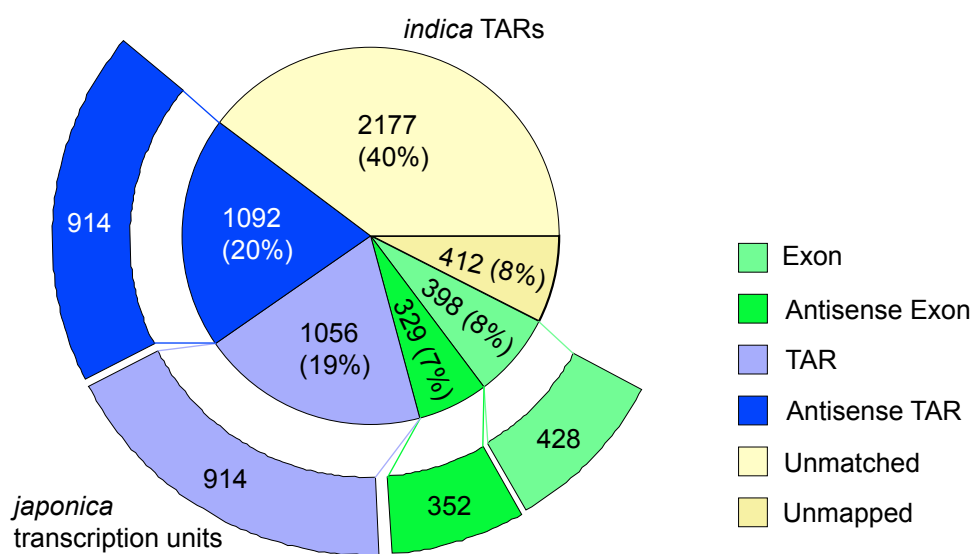

B

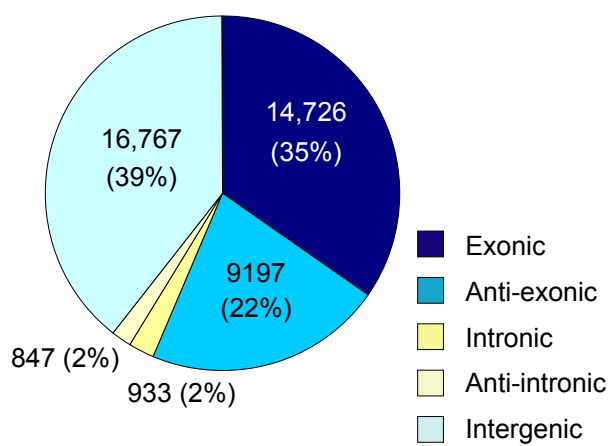

C

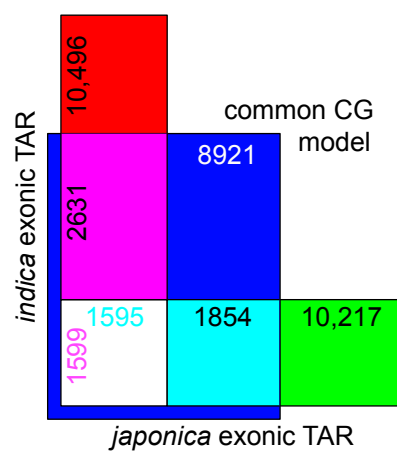

Supplement: Figure S3 — Comparative analysis of TARs. (a), Comparison of 5464 indica TARs with transcription units in japonica. The indica TARs were identified in a previous genome tiling analysis of the indica genome (Ref.2). Exon, indica TARs matched with exons of japonica genes, Antisense Exon, indica TARs mapped to the antisense strand of japonica genes, TAR, indica TARs matched with japonica novel TARs, Antisense TAR, indica TARs mapped to the antisense strand of japonica novel TARs, Unmatched, indica TARs do not match with any japonica transcription unit, Unmapped, indica TARs not mapped to the japonica genome. (b), Classification of 42,470 indica TARs based on their physical relation with annotated indica gene models. The 42,470 TARs were identified by re-analyzing the previous indica genome tiling data (Ref.2) using the same criteria described in Methods. (c), Intersection of exonic TARs with common FL-cDNA supported gene (CG) models. Gene models in both japonica and indica were aligned to rice FL-cDNA to identify the CG models, which were mapped against each other using BLAT. Models were considered common if they overlap >100 bp in the annotated ORFs (Ref.1). Exonic TARs from japonica (green) and indica (red) were matched against the common CG models (blue) and represented by the cyan and magenta color, respectively. TARs matched to the CG models in japonica and indica were compared using BLAT and considered common if they overlap by at least 1 bp (white). (0.25 MB PDF) [file pone.0000294.s003.pdf]

A

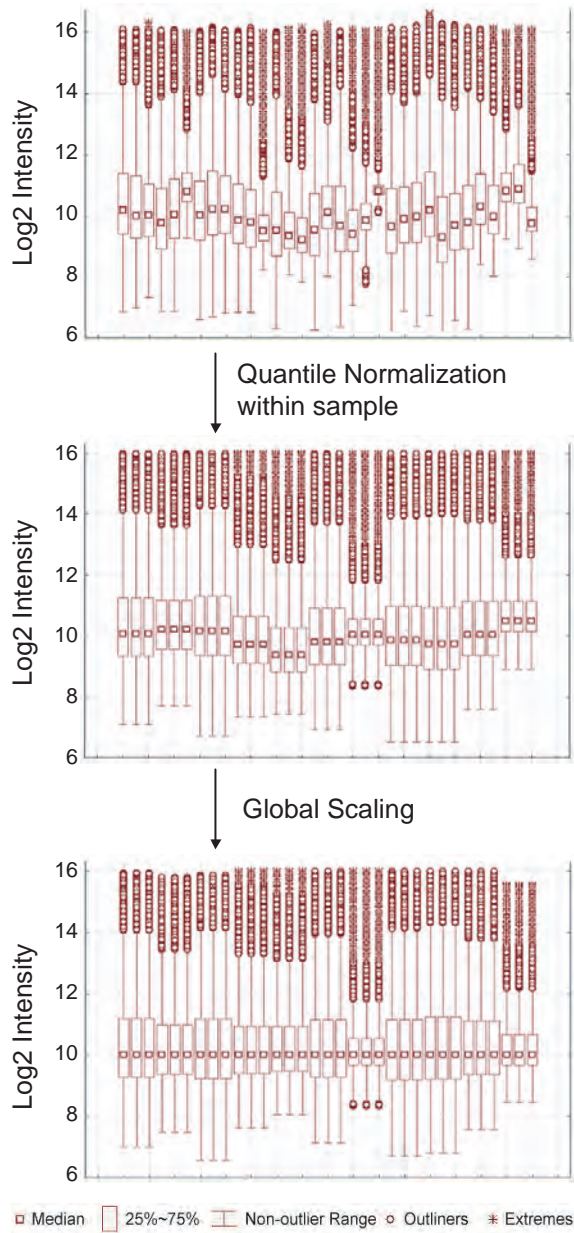

B

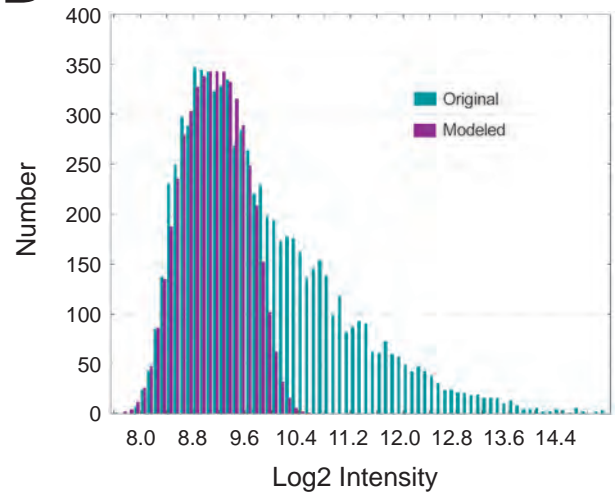

C

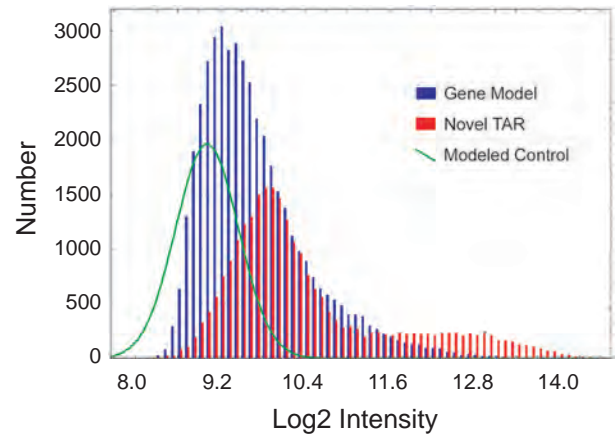

Supplement: Figure S4 — Re-array data processing. (a) Normalization of the re-array hybridization data. Spatial-effect smoothed probe intensities were normalized following a two-step process. First, quantile normalization was performed on the three replicates within each assayed tissue type. Next, a global scaling was carried out to adjust the quantile-normalized intensities to a common baseline. (b) Modeling the 7669 control probes. To determine from the control probes the noise distribution, we used a previously reported modeling process (Ref.1). Assuming the control probes that have very low intensities (1-9 Log2) were primarily noise, we modeled the noise as a normal distribution by mirroring the low-intensity portion of the overall control probe distribution. (c) Transcription threshold determination for individual tissue types. FDR = 0.05, which represents the 95% confidence in the modeled noise distribution, was used as a cutoff for transcription calling. (1.25 MB PDF) [file pone.0000294.s004.pdf]

A

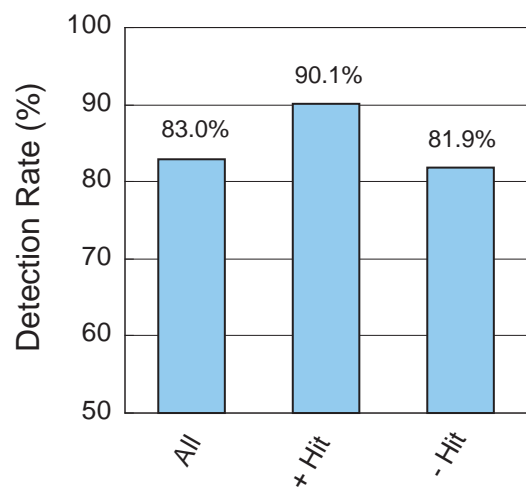

B

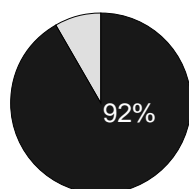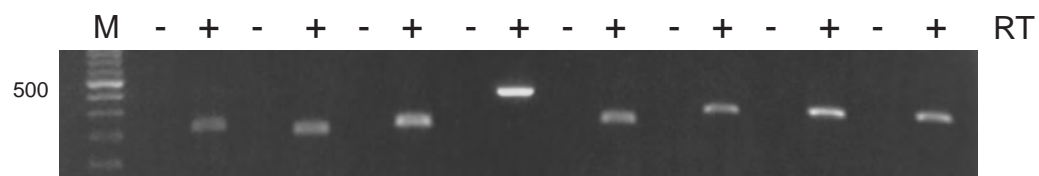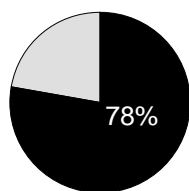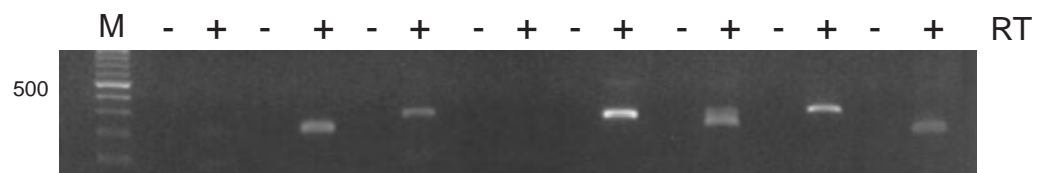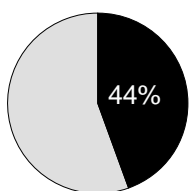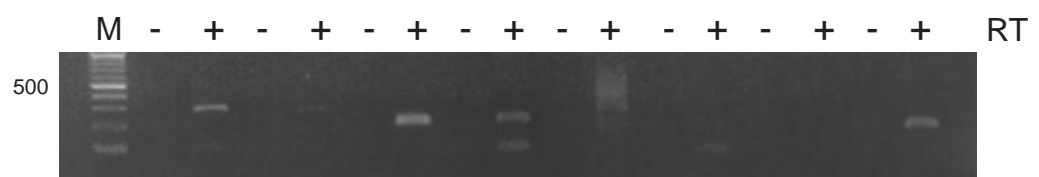

Supplement: Figure S5 — Transcriptional verification of non-exonic TARs. (a) Re-array detection of different groups of TARs. Detection rate was calculated as the percentage of TARs detected in at least one of the 10 assayed tissue types by the re-array. All, all non-exonic TARs; +Hit, TARs match with rice Unigenes; -Hit, TARs do not match with Unigenes. (b) RT-PCR analysis of selected non-exonic TARs. Pie charts indicate the percentage of TARs generated a PCR product of the expected size in an RT-dependent manner. PCR for 36 TARs from each group (108 in total) was performed on reverse transcribed cDNA (+RT) and mRNA (-RT) and resolved on gel side by side. A typical gel from each group is shown. Top, TARs detected by the re-array and have hits against Unigenes; middle, TARs detected by the re-array but have no Unigene hits; bottom, TARs not re-array detected and have no Unigene hits. M, 100 bp molecular weight marker. (0.88 MB PDF) [file pone.0000294.s005.pdf]

A

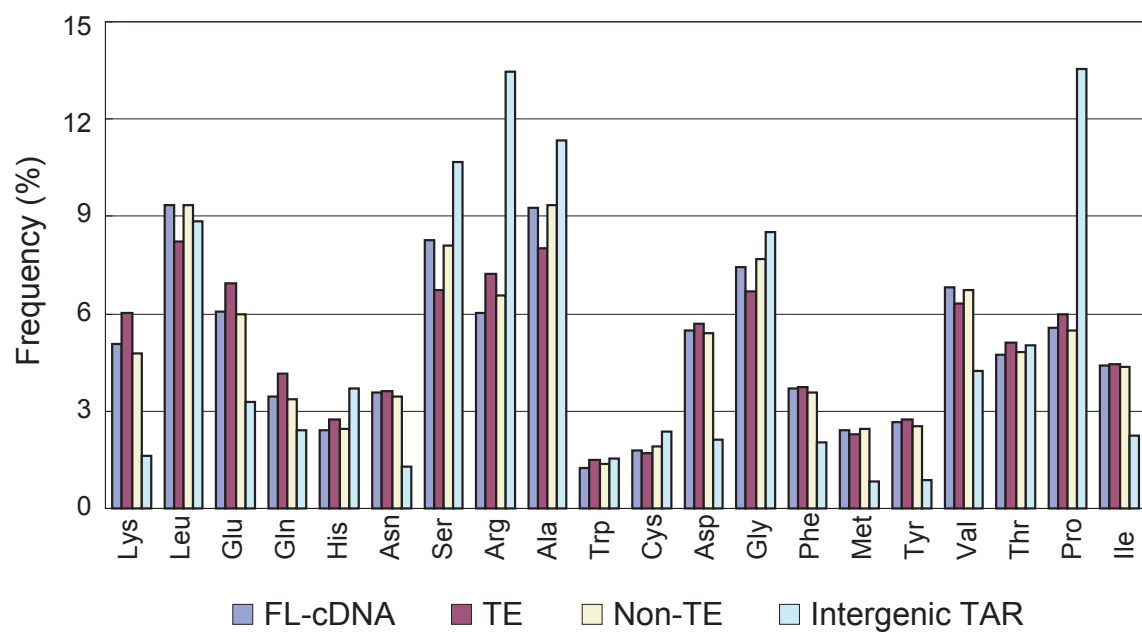

B

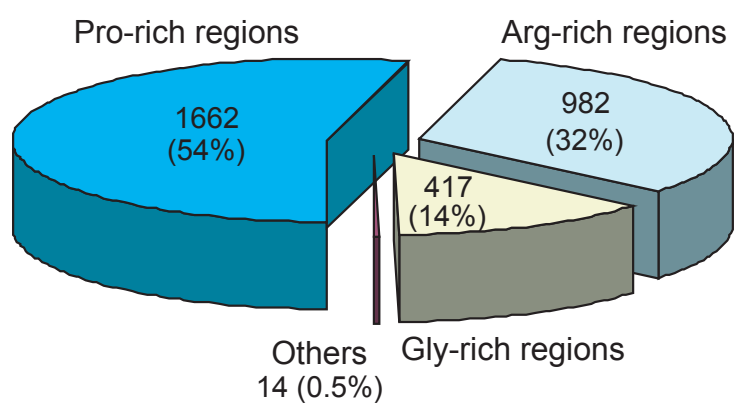

Supplement: Figure S6 — Analysis of the coding potential of intergenic TARs. (a) Frequency of the amino acids contained in the deduced protein sequences of rice FL-cDNA, TE and non-TE gene models, and intergenic TARs. (b) Pie chart analysis of the hits against the ProSite database using deduced polypeptide sequences from the intergenic TARs. (0.22 MB PDF) [file pone.0000294.s006.pdf]

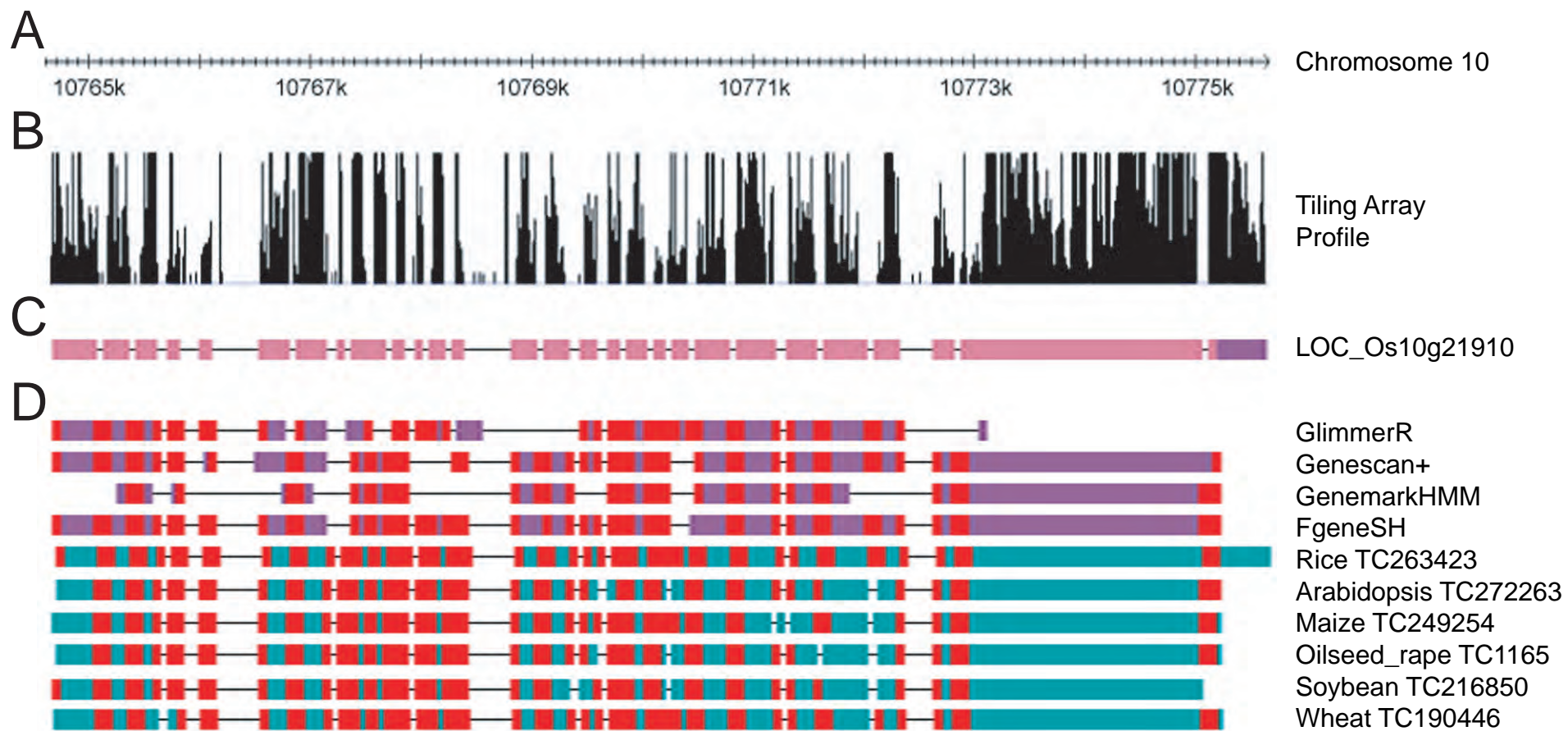

Supplement: Figure S7 — High-resolution tiling array analysis of mRNA target. (a) A region from rice Chromosome 10 represented by the tiling array. (b) Tiling array profile showing each probe aligned to the chromosomal coordinate with its fluorescence intensity depicted as a vertical line. (c) TIGR working gene model from the locus LOC_Os10g21910, which encodes a putative acetyl-coenzyme A carboxylase ACC1A. (d) Gene predictions and experimental evidence supporting the gene model. Red bars indicate significant matches with the working gene model. Data downloaded from the TIGR rice genome annotation database at http://rice.tigr.org/. (1.28 MB PDF) [file pone.0000294.s007.pdf]
